# Putative Microbial Population Shifts Attributable to Nasal Administration of *Streptococcus salivarius* 24SMBc and *Streptococcus oralis* 89a

**DOI:** 10.1007/s12602-018-9488-6

**Published:** 2018-12-07

**Authors:** Roberta De Grandi, Lorenzo Drago, Alessandro Bidossi, Marta Bottagisio, Matteo Gelardi, Elena De Vecchi

**Affiliations:** 1 Laboratory of Clinical Chemistry and Microbiology, IRCCS Orthopedic Institute Galeazzi, Via Galeazzi 4, 20164 Milan, Italy; 2 Laboratory of Clinical Microbiology, Department of Biomedical Sciences for Health, University of Milan, Milan, Italy; 3 Otolaryngology Unit, Department of Basic Medical Science, Neuroscience and Sensory Organs, University of Bari Aldo Moro, Bari, Italy

**Keywords:** Nasal bacteria, 16S RNA gene sequencing, Microbial networks

## Abstract

Changes in bacterial composition of nasal microbiota may alter the host’s susceptibility to several infectious and allergic diseases such as chronic rhinosinusitis and allergic rhinitis. The aim of this study was to evaluate the effects of 1-week administration of a probiotic product, composed by a combination of *Streptococcus salivarius* 24SMBc and *Streptococcus oralis* 89a, on the nostril microbiota. Differences in the nasal microbiota composition were investigated by using a next-generation sequencing approach. A strong and significant decrease in *Staphylococcus aureus* abundance was detected immediately after the bacterial administration. Moreover, comparing the microbial networks of nostril microbiota before and 1 month after the end of treatment, we detected an increase in the total number of both bacterial nodes and microbial correlations, with particular regard to the beneficial ones. Furthermore, a less abundance of microbial genera commonly associated to potential harmful bacteria has been observed. These results suggest a potential ability of *S. salivarius* 24SMBc and *S. oralis* 89a to regulate and reorganize the nasal microbiota composition, possibly favoring those microorganisms that may be able to limit the overgrowth of potential pathogens.

## Introduction

The nasal microbiota is a complex microbial community, composed of several different genera of aerobic and anaerobic microorganisms such as *Staphylococcus* spp., *Corynebacterium* spp., and *Propionibacterium* spp. [1]. Changes in the composition of nasal microbial population may lead to the dysbiosis of nasal microbiota, thereby favoring susceptibility to several inflammatory, infectious, and allergic diseases such as chronic rhinosinusitis, allergic rhinitis, and otitis [2–4]. Till now, systemic antibiotics and anti-inflammatory therapies have been the primary strategies used for the management of these pathological conditions. However, extensive evidences suggest that antibiotics may have limited efficacy [5]. Several studies demonstrated how commensal α-hemolytic streptococci could be used to recover the normal nasopharyngeal flora in children with recurrent otitis media [6–8]. These microorganisms may not only restore the balance between beneficial commensal bacteria and pathogenic species but may also prevent development of antimicrobial resistance due to the intensive use of antibiotics in the treatment of nasal diseases [9]. The use of bacteria as a beneficial approach has been a common practice that is already applied in different fields, such as gastroenterology, gynecology, and dermatology for treatment of functional disorders [10, 11].

Recently, a probiotic product based on the combination of *Streptococcus salivarius* 24SMBc and *Streptococcus oralis* 89a has been developed for direct nasal administration through a vaporizer for the prophylaxis and treatment of chronic and recurrent infections of the upper airways. In this paper, we present the effect of the administration of these two streptococci on the nasal microbiota composition of healthy subjects by evaluating changes in bacterial abundance and microbial correlations in the microbiota network.

## Material and Methods

### Enrollment of Subjects

Twenty healthy volunteers (11 males and 9 females, age 30 ± 5 and 32 ± 4, respectively) participated in this study. They were informed in detail about the purpose of the study and a written consent was obtained from each subject. Exclusion criteria were antibiotic treatment in the previous 2 months and use of any other medical device for treating nasal congestion, such as nasal nebulizers or nasal irrigation devices, and no pets in participant’s homes. The product was based on a mixed dual-species of *S. salivarius* 24SMBc and *S. oralis* 89a in a 98:2 ratio, suspended in a PEG/PPG copolymer (poly-ethylene glycol chain bonded with poly-propylene glycol), and pH 7.00-buffered isotonic solution. Probiotics were administered with two bilateral spray inhalation into each anterior nostril for 1 week, usually in the morning after showering or personal care/washing.

### Sample Collection and DNA Extraction

Sterile swabs in polypropylene tubes (Thermo Fisher, Italy) were used for sampling the mucosal surface from the anterior left nostril (depth 1 cm from the outer edge). From each participant, four nasal swabs were collected as follows: before the probiotic treatment, 1 week after the use of *S. salivarius* 24SMBc and *S. oralis* 89a, 2 weeks after the end of treatment, and 1 month after the end of the probiotic administration. Samples were rapidly frozen at − 80 °C until analysis which began, in any case, within 48 h from sample collection. DNA was extracted using the QIAamp DNA Mini Kit (Qiagen, Italy).

### Library Preparation and Sequencing

Partial 16S rRNA gene sequences were amplified from the extracted DNA using the Ion 16S Metagenomics Kit (Life Technologies, Italy) by two separate PCR reactions using primer set V2, V4, V8 and V3, V6–7, V9. The PCR products were processed to obtain the DNA library using the Ion Plus fragment Library kit (Life Technologies, Italy) and the Ion Xpress Barcode Adapters 1–16 kit (Life Technologies, Italy). Adapter-ligated and nick-repaired DNA was amplified with the following steps: 1 cycle of 25 °C for 15, 72 °C for 5 min, followed by hold at 4 °C. For each step, a cleanup procedure was performed using the Agencourt AMPure XP DNA purification beads (Beckman Coulter Genomics, Bernried, Germany). Each DNA library was eluted in low Tris-EDTA buffer (Life Technologies, Italy). The final DNA concentrations of the purified products were assayed using the Qubit Fluorometer 2.0 (Thermo Fisher Scientific) according to the manufacturer’s instructions. Each sample was adjusted to 26 picomolar DNA concentration. Equal volumes of each library were combined and processed with Ion PGM HI-Q View OT2 Kit and One-Touch ES systems (Life Technologies, Italy) according to the manufacturer’ instructions. Sequencing of the amplicon libraries was performed on a 316 chip using the Ion Torrent Personal Genome Machine (PGM) system and employing the Ion PGM Hi-Q View Seq kit (Life Technologies, Italy). Base calling and run demultiplexing were performed by Torrent Suite 5.1. (Life Technologies, Italy), with default parameters. Data processing was performed using Ion Reporter Software (Life Technologies, Italy) which comprises a suite of bioinformatics tools which automatically provide to add read labels in order to mimic non-demultiplexed data for downstream analysis and concatenating reads into one file. The reads were aligned to the MicroSEQ ID library and to the Greengenes database to achieve rapid and exhaustive bacterial identification with a similarity coverage of 97%. The final output of Ion Reporter Software was the identification and abundance of microorganisms at the phylum, class, order, family, genus, and species levels.

### Statistical Analysis

Statistical analysis and the calculation of the biodiversity indices (Shannon’s, Simpson’s, and Chao’s) were performed using the Vegan 2.4.3 package for R Software V.3.3.1 for Windows. Nonparametric tests based on the Kruskal–Wallis and Wilcoxon rank-sum tests were used to determine the significant differences in *α* diversity and microbial taxa. Adjustment for multiple testing was evaluated with Benjamini-Hochberg FDR correction; *p* values below 0.05 were considered statistically significant. The OTU abundances were used to calculate an adjacency matrix based on the Spearman’s correlation coefficient (*r*_s_) within each bacterial genus to evaluate the strength of a linear association between different bacterial genera. Using Cytoscape _v3.4.0, the adjacency matrix was integrated into a network model for investigating the topological features of the microbial correlations. The microbial network topology was evaluated considering an attribute circle layout and setting the node size on the node’s degree: hubs with small sizes and light colors had low values of node’s degree, hubs with largest number of connections with other microorganisms in the network were considered “central nodes” and highlighted by a diamond shape. Conversely, nodes with less relevant number of connections were defined as “leaves” nodes. Moreover, the strength of a linear association between different bacterial genera was underlined, selecting all edges with an absolute *r*_s_ ≤ 0.60 at a 0.05 significance level. Low values of Spearman’s correlation coefficient were highlighted for edges’ lines for as gray dot lines; differently, connections with 0.80 ≤ *r*_s_ ≤ 0.99, corresponding to a very strong strength of a linear association, were set as solid lines. Finally, positive correlations were highlighted in green, while negative ones were marked in red.

## Results and Discussion

**Fig. 1:**
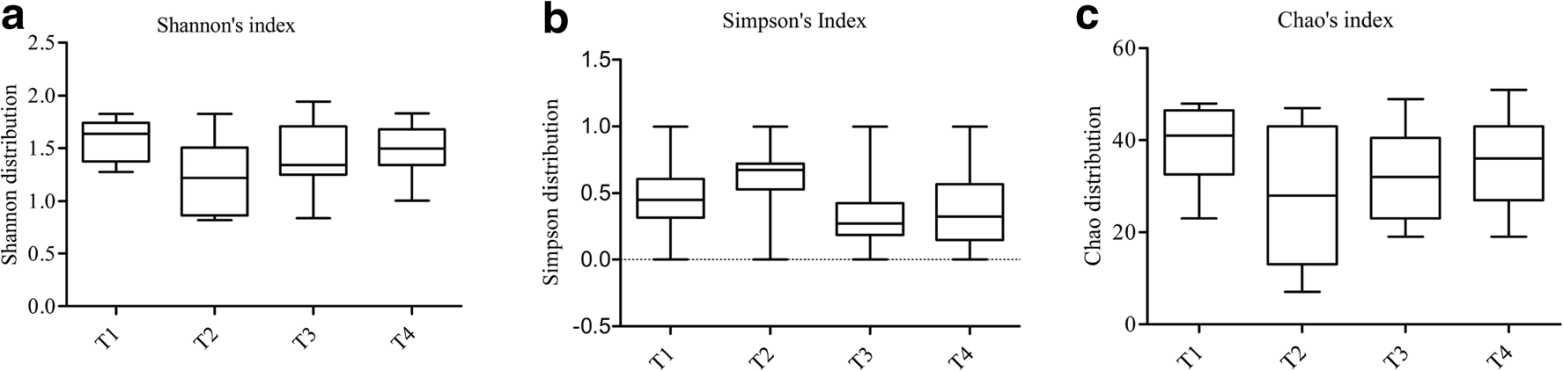
Biodiversity data. The boxplot reported the variation before the probiotic intake (T1), 1 week after the probiotic treatment (T2), 2 weeks after the probiotic intake (T3), and 1 month after the probiotic treatment (T4)

**Fig. 2:**
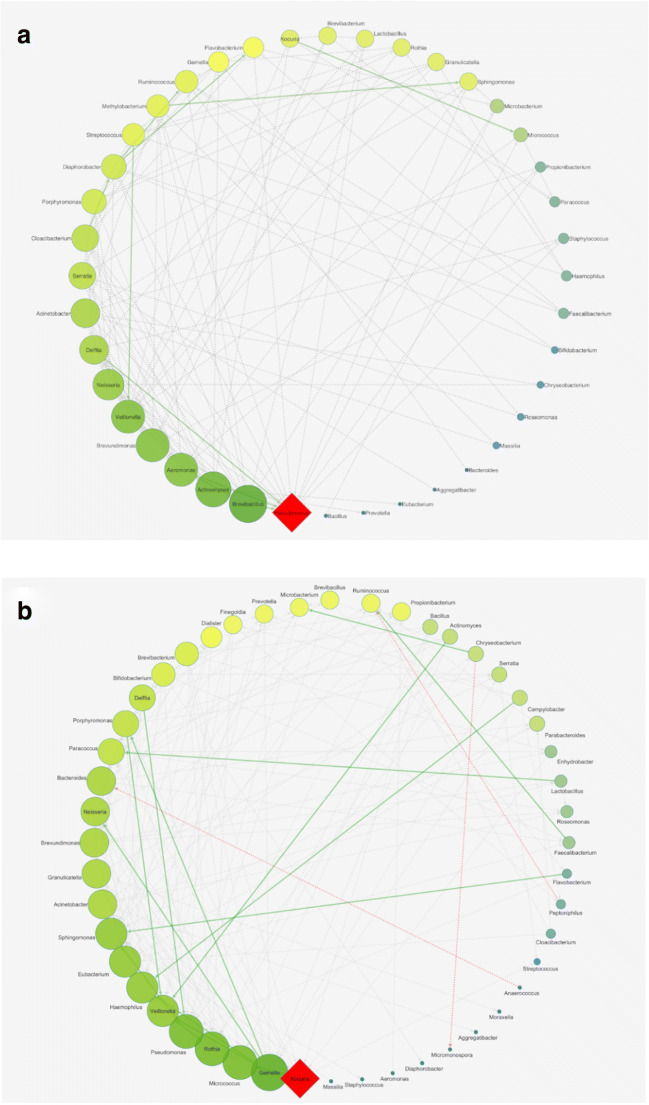
Co-occurrence networks of nasal microbiota before and 1 month after the end of probiotic intake. Node colors from bright to dark represent interaction degrees from low to high of each node linked by other nodes. The node labels are the bacterial genera OTU. A diamond shape was selected to emphasize the node that has the greater number of leaves’ nodes linked to it which was defined as central hub. Gray dot lines were used to set low values of Spearman’ s correlation instead edges with 0.80 ≤ *r*_s_ ≤ 0.98 were highlighted as solid lines. Positive correlations were set in green, while negative ones were marked in red. **a** The bacterial network before the probiotic administration was constructed using a circular layout with 40 spatially co-occurring OTU pairs and 121 total connections. *Pseudomonas* spp. was the central hub of this map. Edges were all positive correlations. **b** The microbial network 1 month after the end of probiotic intake was constructed using 154 edges and 48 spatially co-occurring OTU pairs; of these, 151 were all positive correlations, while three connections were negative (with 0.60 ≤ *r*_s_ ≤ 0.79). *Kocuria* spp. was the central hub of this network as it has the most number of connections

**Fig. 3:**
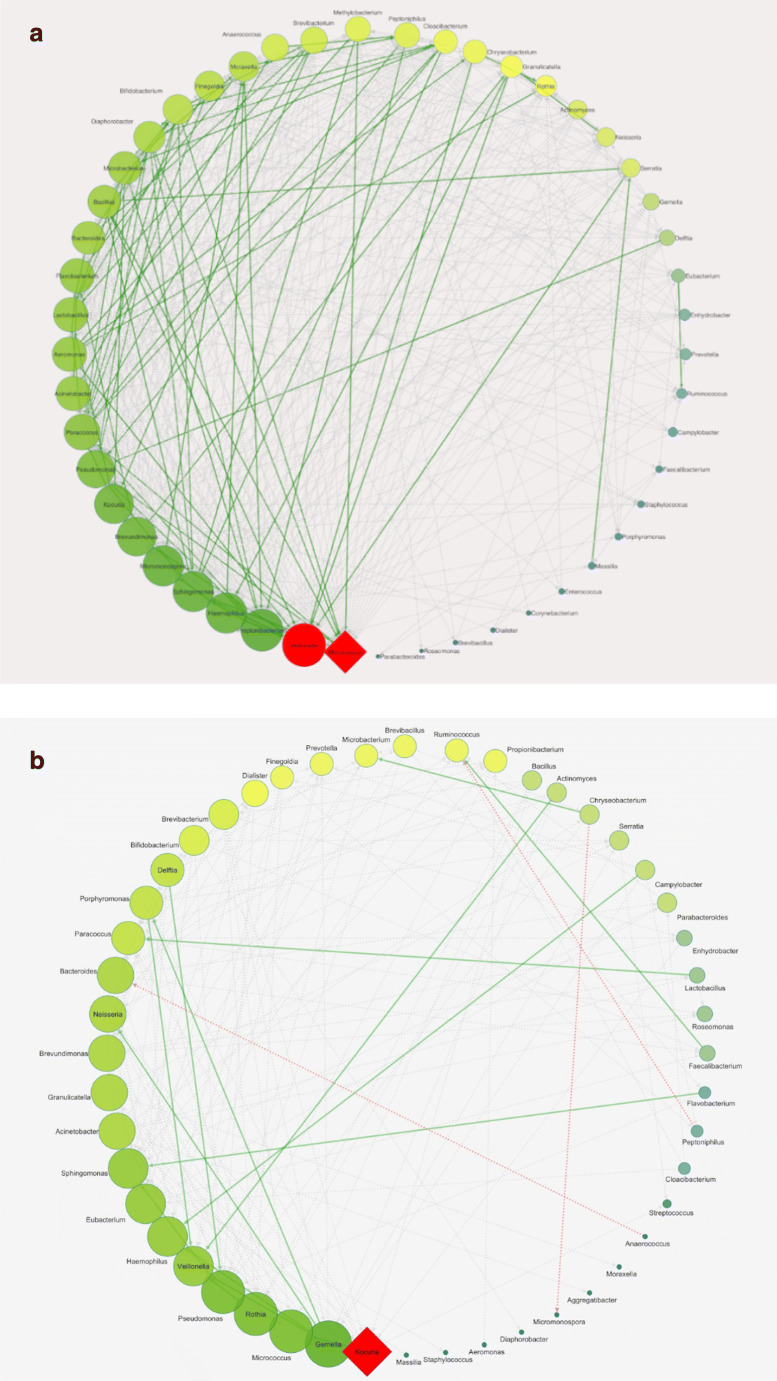
Co-occurrence networks of nasal microbiota immediately after the probiotic intake and 1 month after the end of the nasal streptococci administration. Node colors from bright to dark represent degrees from low to high of each node linked by other nodes. The node labels are the bacterial genera OTU. A diamond shape was selected to emphasize the node that has the greater number of leaves’ nodes linked to it which was defined as central hub. Gray dot lines were used to set low values of Spearman’ s correlation instead edges with 0.80 ≤ *r*_s_ ≤ 0.98 were highlighted as solid lines. Positive correlations were set in green, while negative ones were marked in red. **a** The network investigated immediately after the probiotic intake was generated using 49 spatially co-occurring OTU pairs and 455 total connections. *Microbacterium* spp. was identified as the central hub and the edges are all positive correlations. **b** The bacterial network 1 month after the end of the nasal streptococci administration was constructed using 154 edges and 48 spatially co-occurring OTU pairs; of these, 151 were all positive correlations, while three connections were negative (with 0.60 ≤ *r*_s_ ≤ 0.79). *Kocuria* spp. was the central hub of this network as it has the most number of connections

**Fig. 4:**
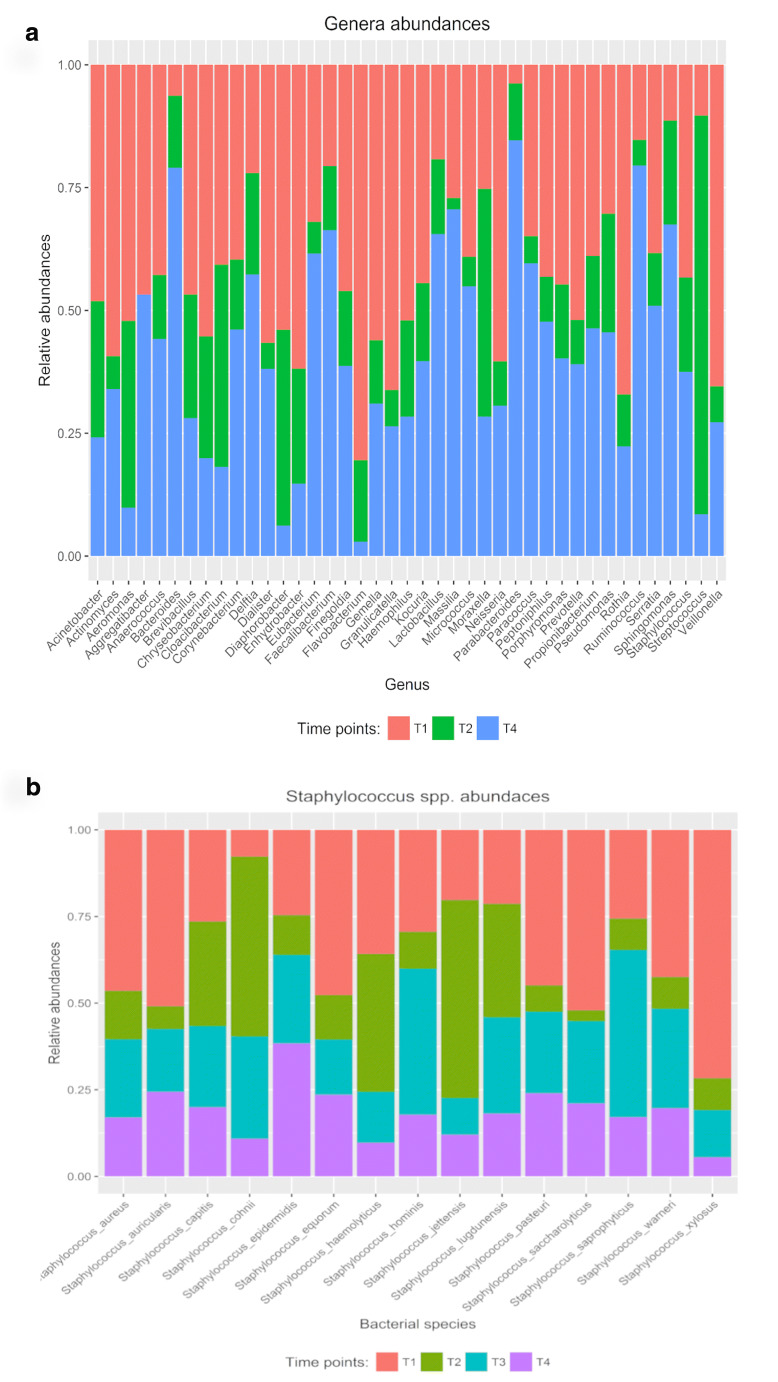
Significant variation in microbial abundances. The histograms reported only the significant variation before the probiotic intake (T1), 1 week after the probiotic treatment (T2), 2 weeks after the probiotic intake (T3), and 1 month after the probiotic treatment (T4). In particular, the panel **a** shows the bacterial genera that are significantly different in the nasal microbiota composition before probiotic administration and after 1 week of treatment with *Streptococcus salivarius* 24SMB and *Streptococcus oralis* 89a (*p* ≤ 0.05). Differently, panel **b** reports only the abundance of *Staphylococcus* species significantly changing during time are reported (*p* ≤ 0.05)

During the experimental time course, no severe side effects were observed in any of the subjects. Immediately after the end of probiotic administration, we observed a significant reduction in the bacterial richness (Fig. 1). This reduction could be due to a potential ability of the two strains to displace the pathogens or to predominate the unwanted microbial species, as previously described for certain lactic acid bacteria that exert antiadhesive and antimicrobial effects against *S. aureus* strains or other opportunistic pathogens colonizing the human intestinal tract [12, 13]. This hypothesis was further suggested by the inverse trend that we observed 1 month after the nasal spray administration, in which the bacterial richness tended to resemble the baseline value, and by the results obtained with the microbial network analysis, in which an increase in number of interactions could be observed, as reported in Fig. 2. Moreover, comparing the microbial maps of the nasal microbiota before and 1 month after the probiotic treatment, we observed an increase in the total number both of bacterial nodes and of microbial correlations, especially considering the positive ones (Fig. 2a, b, respectively). These results may suggest that a reorganization of the nasal microbiota occurs after the administration of the nasal spray. Assuming that the highlighted correlations could reflect potential interaction between different microorganisms, comparison of the microbial networks immediately after the probiotic intake and 1 month after the end of administration evidenced some interesting relationships (Fig. 3a, b, respectively). For example, immediately after the probiotic intake, both *Veillonella* spp. and *Micrococcus* spp. were identified as central hubs (Fig. 3a) but 1 month after the end of the probiotic intake, only *Micrococcus* spp. resulted as the node with the highest number of connections in the bacterial network (Fig. 3b). Shukla et al. described *Veillonella* spp. to be more prevalent in the nasal microbiota of urban non-farmers, who are very similar to our healthy volunteers living in widely urbanized areas [14] and Periasamy and Kolenbrander reported that *Veillonella* species had a central role as early colonizers in establishing multispecies oral biofilm communities [15]. Conversely, micrococci are common human commensals that colonize the skin, the mucosa, and the oropharynx. Some species belonging to the *Micrococcus* genus, such as *Micrococcus luteus* and *Micrococcus superificus*, have been demonstrated to possess a strong inhibition ability towards peptidoglycan biosynthesis and N-glycosylation of proteins [16, 17]. These properties could be responsible of a possible antimicrobial activity of such *Micrococcus* species against Gram-positive bacteria [17]. It could be speculated that probiotic intake is able to favor the microbial interactions of Veillonella spp. and Micrococcus spp., thereby causing these taxa to play a key role in regulating the initial, middle, and late colonizers of the nasal environment or limiting the growth of potential pathogens, such as some species of *Staphylococcus*, which strongly colonize the nasal cavity. Interestingly, after 1 week of probiotic administration, there was a significant increase in *Staphylococcus* spp. abundance (Fig. 4a). However, studying this genus at a deeper taxa level, we observed that the bacterial load of *S. aureus* was subjected to a strong and significant decrease immediately after the probiotic intake (Fig. 4b), especially when compared to that of coagulase-negative staphylococci. A recent study analyzed the nasal staphylococci isolated by bacterial culture techniques for antimicrobial activities and reported that these bacteria produced antimicrobials at an unexpectedly high rate (86%) against numerous nasal bacteria [18]. *Staphylococcus epidermidis* is known to secrete high levels of extracellular serine protease (Esp), which limits *S. aureus* nasal colonization, probably by degrading its surface adhesins or epithelial protein ligands [18]. Similarly, nasal *Staphylococcus lugdunensis* can synthesize an antibacterial compound known as lugdunin that inhibits and counteracts the growth of *S. aureus* [19]. In addition, *Staphylococcus xylosus*, *Staphylococcus warneri*, and *Staphylococcus hominis* have been described as bacteriocin-producing species as they are capable of producing antimicrobial molecules actively in the nasal microbiota [18–20].

Competition by antibiosis may also be one mechanism by which *S. salivarius* 24SMBc and *S. oralis* 89a may carry out their probiotic activity. Bowe et al. reported the in vitro inhibition of *Propionibacterium acnes* through a bacteriocin-like inhibitory substance (BLIS-like substance) produced by a strain of *S. salivarius* [21]. A strong bacteriocin-like inhibitory activity has also been demonstrated for *S. salivarius* 24SMB (DSM 23307), with an inhibitory spectrum mainly targeted against *Streptococcus pneumoniae* and some clinical isolates of *Streptococcus pyogenes* [22]. Moreover, *S. oralis* 89a genome harbors genes encoding for the bacteriocin Colicin V and for tolerance to Colicin E2 [23]. These evidences would lead to hypothesize that *S. salivarius* 24SMBc and *S. oralis* 89a may mediate bacterial species competition in the nasal cavity through distinct direct or indirect mechanisms.

Our data underline the importance to better investigate the specific pathways that regulate the stability or resilience of a bacterial ecosystem to develop strategies of ecological modulation, especially in individuals with dysbiosis or whose microbiota is characterized by a low biodiversity. Investigating the bacterial correlations, based on the microbial abundances, the present study has emphasized the need to integrate the study of the microbial network analysis methods to have a complete and clear characterization of the bacterial populations, as already stated in a previous study [24]. One limitation of this study is the lack of a control group or of a control treatment in the enrolled subjects, to better evaluate the effects on nasal microbiome of probiotics’ administration. This choice was mainly due to the need to exclude allergic subjects and those who did not have consumed antibiotics and probiotics in the period before the study. At the same way, the need of a long washout period between administration of control and probiotic preparations prompted us to choose a pre-dosing sample before administration of the tested probiotics.

Moreover, the study of bacterial interactions by the construction of microbial network is still a little-researched field. Therefore, it is likely that there are still a lot of gaps in the knowledge that need to be filled and that could be useful to support many of the hypothesis arose by the interpretation of the results obtained. For these reasons, the results of the present study need to be taken with considerable caution. To better understand the meaning of the correlations here highlighted, we are currently investigating, by means of in vitro studies, the ability of *S. salivarius* 24SMB and *S. oralis* 89a to interfere with adhesion and biofilm formation of typical upper respiratory tract pathogens. Further investigations could also concern the constructions and analysis of functional networks that, based on producing function-taxonomy link, can better describe the association between the microbial community and its functions. In the future, contextualizing discrete functions of a microbial network will contribute to elucidate the role that specific microbes play on host’s health as well as the potential molecular mechanisms involved in the microbiota-host interaction. A recent study has highlighted that the nasal microbiome of healthy dairy farmers has a greater biodiversity than that of non-farmers living in urban settings, as the farmer individuals are exposed to a more complex microbial environment [14]. Indeed, the interactions with the surrounding environment play a pivotal role in enriching the commensal microbiota and enhancing its interaction with the host’s immune system. For this reason, the use of health-associated microorganisms, such as alpha-hemolytic streptococci, might promote beneficial modulations, especially for those individuals living in urban areas where a decreased microbial biodiversity has been associated with an increased incidence of allergic and inflammatory disorder [25]. In conclusion, our preliminary results highlighted the ability of *S. salivarius* 24SMBc and *S. oralis* 89a to influence the nasal microbiota composition, even though all the observed bacterial changes were not fully characterized. However, a reduction of potential harmful bacteria is an important feature of the two probiotic streptococcal strains and indicates a promising use for future applications in the clinical field.
